# One-hour post-load glucose improves the prediction of cardiovascular events in the OPERA study 

**DOI:** 10.1080/07853890.2021.1902557

**Published:** 2021-03-23

**Authors:** Anni Saunajoki, Juha Auvinen, Aini Bloigu, Olavi Ukkola, Sirkka Keinänen-Kiukaanniemi, Markku Timonen

**Affiliations:** a Center for Life Course Health Research, University of Oulu, Oulu, Finland; b Medical Research Center Oulu, Oulu University Hospital and University of Oulu, Oulu, Finland; c Research Unit of Internal Medicine, Medical Research Center Oulu, Oulu University Hospital and University of Oulu, Oulu, Finland; d Healthcare and Social Services of Selänne, Pyhäjärvi, Finland; e Unit of General Practice, Oulu University Hospital, Oulu, Finland

**Keywords:** Oral glucose tolerance test, dysglycemia, cardiovascular outcomes, prediction

## Abstract

**Background:**

To estimate the ability of fasting, 1-h, and 2-h post-load glucose to predict cardiovascular outcomes.

**Methods:**

We examined a population-based study consisting of 977 middle-aged subjects who underwent an oral glucose tolerance test with glucose values measured at 0, 60, and 120 min. Participants were followed up to 24 years, and cardiovascular outcomes were collected from national registers. Predictive abilities of fasting, 1-h, and 2-h glucose were evaluated alone and in the prediction models with traditional cardiovascular risk factors using Cox proportional hazard models, the likelihood-ratio test, Harrell's concordance index and integrated discrimination improvement.

**Results:**

Cardiovascular endpoint occurred in 222 (22.7%) participants during a median follow-up of 19.8 years. In the prognostic models, 1-h glucose (HR 1.67, 95%CI 1.10–2.53), but not fasting or 2-h glucose, predicted cardiovascular events statistically significantly. In addition, when adding glucose parameters into the model including traditional cardiovascular risk factors, only 1-h glucose improved the predictive ability (LR-test *p*=.046). Finally, 1-h glucose found slightly over 50% more cardiovascular endpoints that were not recognized by fasting or 2-h glucose levels.

**Conclusions:**

Our findings support the earlier ones suggesting that 1-h glucose would be a better long-term predictor of cardiovascular morbidity and mortality than fasting or 2-h glucose.

**KEY MESSAGES**
1. In addition to conventional CV risk factors,1-h but not fasting or 2-h post-load glucoses seems to be an independent predictor of cardiovascular events and seems to improve the predictive ability of the traditional cardiovascular risk model.
2. Elevated 1-hpost-load glucose finds a large number (slightly over 50%)of cardiovascular endpoints that were not recognized by fasting or 2-h post-load glucose levels.
3. One-hour glucose seems to be a better long-term predictor of cardiovascular morbidity and mortality than fasting or 2-h post-load glucose.

## Introduction

Cardiovascular disease is a common comorbidity in participants with type 2 diabetes, and prediction and early identification of hyperglycaemia-associated complications are essential [1,2]. Currently, fasting glucose and the 2-hour oral glucose tolerance test (OGTT) are the standard methods for recognizing these high-risk individuals [3]. Nonetheless, 2-hour plasma glucose (2 h-PG) alone more effectively predicts cardiovascular-related morbidity and mortality than fasting glucose among individuals without previous type 2 diabetes [4–6].

The disadvantages of 2-hOGTT are relatively low reproducibility and sensitivity as well as being considered time-consuming [7]. Recent studies have suggested that a 1-h post-load glucose (1-h PG) level during an OGTT has an even greater ability to predict future type 2 diabetes than 2-h PG among those without type 2 diabetes [8–10]. Furthermore, it has been suggested that the 1-h PG level alone, adjusted with several traditional cardiovascular risk factors, is a capable parameter to predict cardiovascular diseases and all-cause mortality independently [11–13].

Previous works have, however, mainly focussed on the relationship between 1-h PG and cardiovascular morbidity and mortality, but the issue of replacing 2-h PG with 1-h PG to predict cardiovascular events is still controversial. This study was conducted to compare the benefit of fasting, 1-h PG and 2-h PG in addition to traditional cardiovascular risk factors to predict cardiovascular events.

## Materials and methods

### Study population

The OPERA (Oulu Project Elucidating Risk of Atherosclerosis) is a population-based study consisting of middle-aged hypertensive subjects and age- and sex-matched control subjects randomly selected from the registers of Social Insurance Institutes between 1990 and 1993. The study was conducted to determine the occurrence of atherosclerotic cardiovascular diseases and risk factors. The study population and methods used to recruit participants have previously been described in detail [14]. Out of 1200 subjects, 1045 took part in the study including 20 (1.9%) participants with previous myocardial infarction and 22 (2.1%) participants with previous stroke, and the overall participation rate was 87.1%. In this study, we excluded participants with missing glucose values or traditional cardiovascular risk factors (*n* = 10), previously diagnosed type 2 diabetes or the use of glucose-lowering medication (*n* = 58), leaving 977 of the 1200 subjects (81.4%) for the final analysis. Cardiovascular outcomes and mortality of the study participants were followed until the end of 2014without the loss to follow-up for other reasons than death (the rate of non-CVD deaths was 119/997 = 12.2%).The study was approved by the Ethics Committee of the Faculty of Medicine at the University of Oulu according to the principles of the Declaration of Helsinki.

### The definition of variables

All study subjects participated in clinical examinations and laboratory testing in the research laboratory of the Department of Internal Medicine, University of Oulu. The standard 75-g OGTT was performed after an overnight (12-hour) fasting period, and glucose values were determined at 0, 60, and 120 min after glucose intake. At baseline, the glucose values were determined from the whole blood, in accordance with the earlier clinical practice in Finland. Because the glucose measures differ between the whole blood and venous plasma, we used a national correction factor of 1.13 to equate the baseline blood glucose to plasma glucose values. According to WHO criteria, the diagnostic values of plasma glucose concentrations in the OGTT were the following: IFG as fasting glucose level of 6.1 to 6.9 mmol/L and 2-h under 7.8 mmol/L; IGT as fasting glucose under 7.0 mmol/L and 2-h of 7.8 to 11.0 mmol/L; type 2 diabetes as fasting glucose of 7.0 mmol/L or more or 2-h of 11.1 mmol/L or more [3,15]. Large population-based studies have suggested that 1-h PG ≥8.6 mmol/L is a diagnostic criterion for prediabetes [16], and the current meta-analyses showed the optimal cut-off point of 1-h PG ≥11.6 mmol/l to diagnose type 2 diabetes [17]. Total cholesterol, triglycerides, high-density lipoprotein (HDL) and low-density lipoprotein (LDL) were measured as previously described [14]. Glomerular filtration rate(GFR) was estimated by serum creatinine using the CKD-EPI equation.

BMI was calculated as weight (kg) divided by height squared (m^2^). An automatic oscillometric blood pressure recorder (Dinamap, Critikon Ltd) was used to measure blood pressure from the right arm in a sitting position and after at least 5 min of rest. Three measurements were made at 1-minute intervals, and the means of the last two measurements were used in the analyses. The waist circumference was measured at the midpoint between the iliac crest and the lowest rib with the subject breathing normally. All subjects answered a standardized health questionnaire covering family history, physical activity, smoking status, medical history and current medications.

### Outcomes

The outcomes examined were cardiovascular disease or death defined according to the relevant International Classification of Diseases (ICD-8, ICD-9, ICD-10) codes. Coronary heart disease (CHD) was defined as diagnoses I20, I21, I22 [ICD-10] and 410, 4110 [ICD-8/9], coronary artery bypass graft or coronary angioplasty or as I20– I25, I46, R96, R98 [ICD-10] and 410–414, 798 (not 7980 A) [ICD-8/9] as causes of death. CVD was defined as CHD or stroke that included I61, I63 (not I636), I64 [ICD-10] and 431, 4330 A, 4331 A, 4339 A, 4340 A, 4341 A, 4349 A, 436 [ICD-9] or 431 (excluding 43101, 43191), 433, 434, 436 [ICD-8] according to the FINRISK criteria. Cardiovascular outcomes were recorded from the Finnish Causes-of-Death Register and the Hospital Discharge Register.

### Statistical analyses

Background characteristics are presented as mean with standard deviation (SD) or as frequency with percentages. Differences in background characteristics were analysed by using Student’s *t*-test for continuous variables and Pearson's chi-square test for categorical variables. Cox proportional hazard models were conducted to assess the value of different PG measurements (categorized as described above) in predicting cardiovascular events. Prior to model building, we checked the validity of proportional hazard assumptions by graphical assessment using Kaplan-Meier curves. We also assessed multicollinearity among predictors by using variance inflation factor (VIF) and deemed the measures to be in an acceptable range (maximum VIF 1.79). We constructed four hazard estimation models. First model included the traditional risk factors i.e. age, sex, systolic blood pressure, waist circumference, smoking status (never smoker, ex-smoker and current smoker) and LDL-cholesterol. Second, third and fourth model comprised of the first model plus fasting PG, 1-h PG or 2-h PG, respectively. We also implemented all the above-mentioned models with two additional risk factors, i.e. estimated GFR and physical activity (heavy regular, regular, no or irregular). A likelihood-ratio test (LR test) was applied to compare the traditional risk factor model with the other models. We calculated the Harrell’s concordance index (C-index) to quantify the models’ discriminative ability. The improvement in model performance was assessed using the Integrated Discrimination Improvement index (IDI). Bootstrapping (with 1000 resamples) was used to estimate confidence intervals. The level of statistical significance was set at *p*<.05. Statistical analyses were performed using SPSS 24 for Windows (Armonk, NY, USA: IBM Corp.) and R version 3.6.2 (R Foundation for Statistical Computing, Vienna, Austria).

## Results

Baseline characteristics of the study participants are presented in Table 1. Some differences by sex were observed. Men had significantly higher blood pressure, estimated GFR, fasting and 1-h PG as well as cholesterol levels apart from HDL cholesterol (*p*<.05). Men also smoked more often than women but gender disparities in hypertension disorder, BMI, physical activity, 2-h PG levels or prevalence of prediabetes and type 2 diabetes were not observed (Table 1).

**Table 1.** Characteristics of the study population of men and women at baseline.

	Men	Women	*p* Value
Study population, *n* (%)	482 (49.3)	495 (50.7)	
Age (years)	50.6 ± 6.0	51.7 ± 6.0	<.05
Current smoking, *n* (%)	158 (32.8)	131 (26.5)	<.001
BMI (kg/m^2^)	27.7 ± 4.0	27.3 ± 5.0	.175
Waist circumference (cm)	96.7 ± 10.4	83.3 ± 11.5	<.001
Fasting plasma glucose (mmol/L)	5.2 ± 1.1	5.1 ± 1.1	<.05
1-h post-load glucose (mmol/L)	8.0 ± 3.1	7.3 ± 3.1	<.001
2-h post-load glucose (mmol/L)	6.3 ± 2.6	6.5 ± 2.6	.199
NGT, *n* (%)	383 (79.5)	396 (80.0)	.809
IFG, *n* (%)	14 (2.9)	11 (2.2)	
IGT, *n* (%)	64 (13.3)	62 (12.5)	
Screen detected diabetes, *n* (%)	21 (4.4)	26 (5.3)	
Total cholesterol (mmol/L)	5.8 ± 1.0	5.6 ± 1.0	<.05
HDL cholesterol (mmol/L)	1.2 ± 0.3	1.5 ± 0.4	<.001
LDL cholesterol (mmol/L)	3.7 ± 0.9	3.4 ± 0.9	<.001
Triglycerides (mmol/L)	1.7 ± 0.9	1.4 ± 0.8	<.001
Systolic blood pressure (mmHg)	151 ± 21	144 ± 22	<.001
Diastolic blood pressure (mmHg)	92 ± 11	86 ± 12	<.001
Hypertensive disorder, *n* (%)	235 (48.8)	249 (50.3)	.629
GFR-CKD-EPI (ml/min/1.73 m^2^)	89.0 (14.4)	79.8 (14.4)	<.001
Physical activity, *n* (%)			.097
Heavy regular	137 (29.2)	173 (35.3)	
Regular	164 (35.0)	147 (30.0)	
No or irregular	168 (35.8)	170 (34.7)	

Continuous variables are presented as mean ± SD. Categorical variables are presented as counts and percentages. NGT: normal glucose tolerance; IFG: impaired fasting glucose; IGT: impaired glucose tolerance.

The composite CVD endpoint occurred in 222 (22.7%) participants during a median follow-up of 19.8 ± 5.7 years of which 35 (15.8%) had fatal myocardial infarction or stroke. Table 2 shows the characteristics of the participants with and without cardiovascular morbidity and/or mortality. The mean values of the CVD risk factors – including age, BMI, waist circumference, LDL cholesterol, blood pressure, fasting and post-load glucose values – were significantly higher among participants with a CVD endpoint. The prevalence of smoking and hypertension disorder was also significantly higher among the CVD group, whereas estimated GFR or physical activity did not associate with CVD outcomes (Table 2).

**Table 2.** Clinical characteristics of participants with and without cardiovascular morbidity and/or mortality.

	No cardiovascular diseases	Cardiovascular morbidity and/or mortality	*p* Value
Study population, *n* (%)	755 (77.3)	222 (22.7)	
Sex, *n* (% male)	329 (43.6)	153 (68.9)	<.001
Age (years)	50.7 ± 6.0	52.5 ± 5.9	<.001
Current smoking, *n* (%)	211 (27.9)	78 (35.1)	<.01
BMI (kg/m^2^)	27.3 ± 4.6	28.2 ± 4.2	<.01
Waist circumference (cm)	88.7 ± 12.9	94.2 ± 11.5	<.001
Fasting plasma glucose (mmol/L)	5.1 ± 0.9	5.4 ± 1.6	<.01
1-h post-load glucose (mmol/L)	7.4 ± 2.9	8.5 ± 3.5	<.001
2-h post-load glucose (mmol/L)	6.3 ± 2.3	7.0 ± 3.4	<.01
NGT, *n* (%)	622 (82.4)	157 (70.7)	<.01
IFG, *n* (%)	16 (2.1)	9 (4.1)	
IGT, *n* (%)	85 (11.3)	41 (18.5)	
Screen detected diabetes, *n* (%)	32 (4.2)	15 (6.8)	
Total cholesterol (mmol/L)	5.6 ± 1.0	6.0 ± 1.2	<.001
HDL cholesterol (mmol/L)	1.4 ± 0.4	1.2 ± 0.4	<.001
LDL cholesterol (mmol/L)	3.5 ± 0.9	3.8 ± 1.0	<.001
Triglycerides (mmol/L)	1.4 ± 0.8	1.9 ± 1.1	<.001
Systolic blood pressure (mmHg)	146 ± 22	153 ± 21	<.001
Diastolic blood pressure (mmHg)	88 ± 12	92 ± 12	<.001
Hypertensive disorder, *n* (%)	359 (47.5)	125 (56.3)	<.05
GFR-CKD-EPI (ml/min/1.73 m^2^)	84.3 (15.1)	84.6 (15.1)	.803
Physical activity, *n* (%)			.602
Heavy regular	233 (31.5)	77 (35.0)	
Regular	244 (33.0)	67 (30.5)	
No or irregular	262 (35.5)	76 (34.5)	

Continuous variables are presented as mean ± SD. Categorical variables are presented as counts and percentages. NGT: normal glucose tolerance; IFG: impaired fasting glucose; IGT: impaired glucose tolerance.

The risk of CVD events was estimated by traditional cardiovascular risk factors and three glucose measures in four separate models. The first model included the traditional risk factors, i.e. age, sex, hypertension disorder, waist circumference, smoking status and LDL-cholesterol. An increased risk of cardiovascular events was found in men, current smokers and among participants at an older age, hypertensive disorder and elevated LDL cholesterol. However, no association was observed between CVD endpoints and waist circumference and ex-smokers. When fasting glucose (categorized as ≤6.0, 6.1–7.0 and ≥7.0 mmol/L) was included in the first model, the prediction of CVD outcomes was not improved (LR-test compared to Model I *p*=.113). Adding of 1-h PG (categorized as <8.6, 8.6–11.5 and ≥11.6 mmol/L) to the first model provided a significant improvement in predicting CVD events (LR-test compared to Model I *p*=.046), and 1-h PG ≥11.6 mmol/L was a significant predictor of CVD morbidity and mortality (HR 1.67, 95% CI 1.10–2.53). Conversely, adding 2-h PG (categorized as <7.8, 7.8–11.0 and ≥11.1 mmol/L)to the first prediction model, 2-h PG was not an independent predictor and did not provide the model improvement (LR-test compared to Model I *p*=.193) (Table 3). The discriminative ability was quite good, but comparable for all models (range of C-index 0.695 (95%CI 0.664–0.726) − 0.699 (95%CI 0.668–0.730)). According to IDI values, the improvement in model performance did not differ significantly between the first model and the other models (IDI values for the second, third and fourth model were 0.008 (95%CI −0.002-0.034), −0.007 (95%CI −0.048–0.026) and −0.003 (95%CI −0.022–0.017), respectively). Furthermore, after adding two additional cardiovascular risk factors i.e. estimated GFR and physical activity (heavy regular, regular, no or irregular) to the all four previous models, the results were similar and neither of the new confounders was statistically significant.

**Table 3.** Hazard ratio of cardiovascular outcomes for traditional cardiovascular risk factors, fasting, 1-h PG and 2-h PG.

	Model I HR (95 % Cl)	Model II HR (95 % Cl)	Model III HR (95 % Cl)	Model IV HR (95 % Cl)
Age (years)	1.06 (1.03–1.08)	1.06 (1.03–1.08)	1.06 (1.03–1.08)	1.05 (1.03–1.08)
Sex	2.13 (1.53–2.97)	2.24 (1.60–3.12)	2.25 (1.61–3.13)	2.22 (1.59–3.10)
Hypertensive disorder (over 140/90 mmHg or medication)	1.01 (1.00–1.01)	1.01 (1.00–1.01)	1.01 (1.00–1.01)	1.01 (1.00–1.01)
Waist circumference (cm)	1.01 (1.00–1.02)	1.01 (0.99–1.02)	1.00 (0.99–1.02)	1.01 (0.99–1.02)
Smoking status				
Never smoking				
Ex-smoker	1.11 (0.78–1.57)	1.13 (0.79–1.60)	1.11 (0.78–1.58)	1.12 (0.79–1.60)
Current smoker	1.60 (1.16–2.20)	1.62 (1.17–2.22)	1.64 (1.19–2.25)	1.64 (1.19–2.26)
LDL cholesterol (mmol/L)	1.27 (1.10–1.48)	1.26 (1.08–1.46)	1.28 (1.10–1.48)	1.27 (1.09–1.48)
Fasting glucose (mmol/L)				
≤6.0				
6.1-7.0		1.53 (0.95–2.47)		
≥ 7.0		1.79 (0.84–3.84)		
1-h PG (mmol/L)				
< 8.6				
8.6-11.5			1.29 (0.94–1.79)	
≥ 11.6			1.67 (1.10–2.53)	
2-h PG (mmol/L)				
< 7.8				
7.8-11.0				1.32 (0.92–1.89)
≥ 11.1				1.48 (0.84–2.60)

Model I: traditional cardiovascular risk factors.

Model II: traditional cardiovascular risk factors and fasting plasma glucose, LR-test compared to Model I *p*=.113.

Model III: traditional cardiovascular risk factors and 1-h PG, LR-test compared to Model I *p*=.046.

Model IV: traditional cardiovascular risk factors and 2-h PG, LR-test compared to Model I *p*=.193.

In the further analyses, we evaluated the additional benefit of 1-h PG to fasting and 2-h PG levels to predict cardiovascular morbidity and mortality. In total, 222 of 922 participants (22.7%) had CVD endpoints during the follow-up time and altogether, 65 (65/222 = 29.3%, 95%CI 23.3–35.3%) endpoints were found by abnormal fasting (≥6.1 mmol/l) and/or 2-h PG (≥7.8 mmol/l) levels. Also, 1-h PG with a cut-off point ≥8.6 mmol/L among normoglycemic (<6.1 mmol/l and 2-h PG <7.8 mmol/l) participants found an additional 35 other CVD endpoints, which was statistically significantly more than without using 1-h glucose (65 endpoints) (100/222 = 45.0%, 95% CI 38.5–51.6%, *p*<.001). An abnormal 2-h PG level (≥7.8 mmol/l) recognized five out of 100 (5/100 = 5.0%) CVD endpoints that were not found by abnormal fasting (≥6.1 mmol/l) or 1-h PG (≥8.6 mmol/l) levels (Table 4).

**Table 4.** Prediction of cardiovascular outcomes by fasting, 1-h PG and 2-h PG levels.

	1-h Glucose <8.6 mmol/l		1-h Glucose 8.6–11.5 mmol/l		1-h Glucose ≥11.6 mmol/l	
	All, *n* (%)	CVD endpoint, *n* (%)	All, *n* (%)	CVD endpoint, *n* (%)	All, *n* (%)	CVD endpoint, *n* (%)
Normal glucose, *n* (%)	637 (81.8)	122 (19.2)	129 (16.6)	33(25.6)	13 (1.7)	2 (15.4)
Fasting glucose ≥6.1 mmol/l, *n* (%)	5 (19.2)	1 (20.0)	17 (65.4)	7 (41.2)	4 (15.4)	2 (50.0)
2-h Glucose ≥7.8 mmol/l, *n* (%)	26 (21.7)	5 (19.2)	61 (50.8)	18 (29.5)	33 (27.5)	13 (39.4)
Fasting glucose ≥6.1 mmol/l and 2-h glucose ≥7.8 mmol/l, *n* (%)	2 (3.8)	1 (50.0)	9 (17.3)	3 (33.3)	41 (78.8)	15 (36.6)

## Discussion

An elevated 1-h PG level has been previously associated with cardiovascular outcomes and mortality [13,18,19]. Our result shows that 1-h PG was an independent and better predictor of cardiovascular morbidity and mortality than fasting or 2-h PG levels and found slightly over 50% more CVD endpoints that were not recognized by fasting or 2-h plasma glucose levels. The shortened 1-h OGTT provide the predictive equivalence for cardiovascular outcomes as the 2-h OGTT. In addition, in the prognostic models with traditional cardiovascular risk factors, the addition of 1-h PG, but not fasting or 2-h PG levels, improved the predictability of CVD endpoints. Furthermore, only 1-h PG was an independent predictor of CVD outcomes in the prediction models.

A recent review of the literature has concluded that the postprandial glucose values are the main predictors of future CVD incidence, even in those without type 2 diabetes [4–6,20].Our study confirms the previous findings and highlights the significance of 1-h PG as the strongest determinant of CVD events and mortality. Such predictive capacity of 1-h PG was previously supported by the Malmö Preventive Project. They also showed that 1-h PG is an independent predictor of CVD outcomes after adjusting for other cardiovascular risk factors, which is in line with our study [12]. However, their study population included only middle-aged men. A few more studies have conducted the ability of 1-h PG ≥8.6 mmol/L among normoglycemic participants to predict future cardiovascular outcomes [10,18,21]. The Finnish Diabetes Prevention Study with prediabetic participants found the association of 1-h PG and 2-h PG levels with cardiovascular events among participants with IGT, but only 2-h PG remained an independent risk factor in their pairwise comparisons [15].

As revealed by previous studies, participants with NGT and 1-h PG ≥8.6 mmol/L have impaired b-cell function, lower insulin sensitivity and unfavourable inflammatory and cardiovascular risk profile and are thus at increased risk of developing CVD events [10,22–24]. In –our study, 1-h PG found over one-third of cardiovascular events that would not have been identified with fasting or 2-h PG levels. However, an increased 2-h PG level recognized only 5% of CVD events that were not identifiable by fasting or 1-h PG levels. It appears that participants with a high risk of developing CVD events could be recognized by elevated 1-h PG before progressing to IGT, type 2 diabetes or cardiovascular complications. The shortened 1-h OGTT is more time-saving and cost-effective than a 2-h OGTT and may be therefore considered as an alternative predictive tool for evaluating the risk of CVD events.

There are some limitations to our study. The number of participants in the present study and in the certain subgroup analyses was relatively small, and therefore the estimated confidence intervals for hazard rations were relatively wide. It is also noteworthy that the definition of type 2 diabetes has changed over the past decades, and therefore we were not able to evaluate the predictive ability of HbA1c compared to fasting, 1-h PG and 2-h PG levels in our prediction models of cardiovascular outcomes. However, the previous cross-sectional study suggested that the increased1-h PG level is associated with CVD outcomes, although the HbA1c concentration is in the normal range (HbA1c <5.7%) [11]. The advantages of our study include a relatively long duration of follow-up time and the random sampling method of study participants.

In conclusion, the present study confirms that 1-h PG is a better long-term predictor of cardiovascular morbidity and mortality than fasting or 2-h PG levels among participants without previously diagnosed type 2 diabetes. More prospective studies are needed to confirm our results.

## Data Availability

The datasets generated and/or analyzed during the current study are available from the corresponding author upon reasonable request.
